# Temporal changes in characteristics, incidence, and mortality in patients undergoing surgical intervention for mitral stenosis

**DOI:** 10.1038/s41598-024-74807-5

**Published:** 2024-10-16

**Authors:** Hans T. Due, Jeppe K. Petersen, Daniel E. Meulengracht, Morten H. Smerup, Michael R. Schmidt, Lars Køber, Emil Fosbøl, Lauge Østergaard

**Affiliations:** 1 Rigshospitalet, Hjertecentret, Blegdamsvej 9, 2100 Copenhagen, Denmark; 2 Bispebjerg-Frederiksberg Hospital, Copenhagen, Denmark

**Keywords:** Cardiology, Interventional cardiology

## Abstract

To examine temporal changes in patient characteristics, the incidence rate of surgical intervention, and the three-year mortality rate for patients undergoing surgical intervention for mitral valve stenosis (MS) from 2001 to 2021 in Denmark. Utilizing Danish nationwide registries, we identified all adult patients undergoing first-time surgical intervention for MS (2001–2021). Temporal changes in the incidence rate of surgical intervention across calendar periods were investigated (2001–2005, 2006–2010, 2011–2015, and 2016–2021). Using Kaplan-Meier estimates, we computed temporal changes in three-year all-cause mortality across calendar periods. With multivariable Cox regression analysis, we assessed the adjusted rates of mortality. We included 256 patients (median age 60.3 years, 31.3% males). A stepwise decrease in the burden of chronic heart failure was identified (2001–2005: 54%, and 2016–2021: 20%, p-value < 0.001 for trend), while the burden of atrial fibrillation seemed stable (2001–2005: 51%, and 2016–2021: 46%, p-value = 0.27 for trend). The proportion of patients with rheumatic MS was 64% in 2001–2005 and 33% in 2016–2021, p-value < 0.001 for trend. 28 patients (10.9%) had mitral valve surgery prior to the first-time surgical intervention for MS. A stepwise decrease in the incidence rate of surgical intervention was observed: 3.3 cases per million person-years in 2001–2005 and 2.2 in 2016–2021. The incidence of patients diagnosed with first-time MS remained stable over calendar periods (2001–2005: 54.5 incidences per million person-years, and 2016–2021: 41.9, p-value = 0.46 for trend). The in-hospital mortality was 8.6% and the three-year mortality after surgical discharge was 9.0%, and we found no difference over calendar periods in either crude estimates or adjusted analysis. From nationwide data across three decades, we identified that the incidence of surgical interventiondecreased significantly within the past 20 years, while all-cause mortality remained stable. The burden of heart failure decreased, while the burden of atrial fibrillation remained stable over the study period.

**Supplementary Information:**

The online version contains supplementary material available at 10.1038/s41598-024-74807-5.

## Introduction

Significant mitral valve stenosis (MS) is associated with a poor prognosis, and the only treatment modality for improvement is surgical. For several decades the incidence of MS has been shown to be decreasing in the Western world, however globalization and the movement of population groups across geographic regions urge high-income countries to continue monitoring this disease with a high burden of morbidity and mortality^1–6^.

Surgical intervention is used to treat moderate to severe mitral stenosis^7^ and is thought to be rare in the Western world, but contemporary data assessing the temporal trends to underline this are sparse and especially on an unselected national scale. In asymptomatic patients with rheumatic MS, the 10-year survival rate is around 80%^8^, while the 10-year survival rate in symptomatic patients has been shown to be as low as 15–20%^9–11^ and is associated with cardiac morbidity such as atrial fibrillation (AF) and heart failure (HF)^9^. The incidence of MS in industrial countries has been estimated at 1.0–3.6 per 100,000 inhabitants^9,12^, but is notably higher in developing countries^9^. The temporal changes in the incidence of patients undergoing surgical intervention for MS in the Western world remain unclear, as no larger multicentre study and no nationwide cohorts have looked at this issue in detail previously. These data are important to set an epidemiologic benchmark for a rare yet lethal disease with sparse contemporary data.

This Danish nationwide cohort study set out to investigate the extent of patients undergoing mitral valve surgery for MS, patient characteristics, and three-year mortality following repair (balloon valvotomy included) and replacement of the mitral valve.

## Results

### Study population

**Fig. 1:**
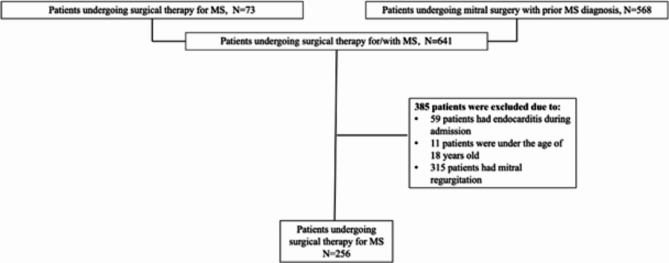
Flow chart of the study population. This flow chart illustrates the population selection process.

From 2001 until 2021, 256 patients had surgical intervention for MS (see selection process in Fig. 1). Table 1 displays patient characteristics according to the pre-specified calendar periods. Of those, 192 patients (75%) underwent replacement (65.2% mechanical valve replacement and 34.8% biological valve replacement), and 64 patients (25%) underwent repair of the mitral valve with a significant trend for less repair in late calendar periods (*p* = 0.051 for trend). Twenty-four patients (9.4%) had concomitant coronary artery bypass graft surgery, and 31 patients (12.1%) had concomitant aortic valve replacement with no clear trend across calendar periods (*p* = 0.21 and 0.20 for trend, respectively). Between 15% and 28% underwent mitral balloon valvotomy over calendar periods with no clear trend (*p* = 0.089). 19 (7.3%) patients had a diagnosis of Grown-Up Congenital Heart disease (GUCH) prior to surgery, and 7 of these patients had congenital mitral valve disease.

**Table 1:** Baseline characteristics for patients undergoing surgical intervention for mitral stenosis by calendar periods.

	Calendar period 2001–2005 *N* = 70			Calendar period 2006–2010 *N* = 78			Calendar period 2011–2015 *N* = 47			Calendar period 2016–2021 *N* = 61		
Demographics												
Male sex (%)	20	(28.6%)		25	(32.1%)		14	(29.8%)		21	(34.4%)	
Age median years (IQR)	57.7	(45.0–69.0)		60.8	(47.2–71.6)		62.7	(44.0-71.4)		59.7	(44.0–69.0)	
Duration of admission												
Median days	12.0	(6.0–18.0)		12.5	(9.0–23.0)		12.0	(6.0–17.0)		11.0	(7.0–18.0)	
Diagnosis of rheumatic mitral valve disease prior to surgical intervention for MS												
	45	(64.3%)		43	(55.1%)		24	(51.1%)		20	(32.8%)	
Type of mitral valve surgery												
Repair	21	(30.0%)		19	(24.4%)		15	(31.9%)		9	(14.8%)	
Replacement	49	(70.0%)		59	(75.6%)		32	(68.1%)		52	(85.2%)	
Balloon mitral valvotomy												
	20	(28.6%)		12	(15.4%)		13	(27.7%)		9	(14.8%)	
Other surgical procedures performed on the day of MS surgery												
CABG	6	(8.6%)		11	(14.1%)		3	(6.4%)		4	(6.6%)	
Aortic valve surgery	11	(15.7%)		10	(12.8%)		< 3			8	(13.1%)	
Tricuspid valve surgery	3	(4.3%)		< 3			4	(8.5%)		5	(8.2%)	
Prior cardiac surgery												
	< 3			7	(9.0%)		5	(10.6%)		3	(4.9%)	
Other heart valve diseases												
Aortic regurgitation	10	(14.3%)		13	(16.7%)		8	(17.0%)		17	(27.9%)	
Aortic stenosis	8	(11.4%)		8	(10.3%)		7	(14.9%)		5	(8.2%)	
Comorbidities												
Stroke	8	(11.4%)		9	(11.5%)		7	(14.9%)		11	(18.0%)	
Bleeding	6	(8.6%)		12	(15.4%)		5	(10.6%)		7	(11.5%)	
Heart failure	38	(54.3%)		28	(35.9%)		18	(38.3%)		12	(19.7%)	
Hypertension	35	(50.0%)		45	(57.7%)		21	(44.7%)		30	(49.2%)	
Ischaemic heart disease	23	(32.9%)		28	(35.9%)		18	(38.3%)		17	(27.9%)	
Atrial fibrillation	36	(51.4%)		35	(44.9%)		21	(44.7%)		28	(45.9%)	
COPD	12	(17.1%)		13	(16.7%)		10	(21.3%)		11	(18.0%)	
Liver disease	4	(5.7%)		< 3			< 3			< 3		
Chronic renal failure	5	(7.1%)		3	(3.8%)		6	(12.8%)		9	(14.8%)	
Diabetes	10	(14.3%)		9	(11.5%)		6	(12.8%)		12	(19.7%)	
Malignancy	7	(10.0%)		3	(3.8%)		6	(12.8%)		12	(19.7%)	
Medications												
Calcium channel blockers	14	(20.0%)		12	(15.4%)		6	(12.8%)		8	(13.1%)	
RAS inhibitors	15	(21.4%)		24	(30.8%)		11	(23.4%)		21	(34.4%)	
Loop diuretics	40	(57.1%)		38	(48.7%)		22	(46.8%)		25	(41.0%)	
Aspirin	14	(20.0%)		25	(32.1%)		13	(27.7%)		8	(13.1%)	
OAC	47	(67.1%)		43	(55.1%)		22	(46.8%)		37	(60.7%)	
Statin	14	(20.0%)		30	(38.5%)		17	(36.2%)		30	(49.2%)	
Beta blockers	22	(31.4%)		28	(35.9%)		16	(34.0%)		29	(47.5%)	
Digoxin	27	(38.6%)		20	(25.6%)		8	(17.0%)		8	(13.1%)	
Geographic region												
Denmark	43	(61.4%)		50	(64.1%)		31	(66.0%)		41	(67.2%)	
Europe (DK excluded)	< 3			3	(3.8%)		< 3			5	(8.2%)	
Asia	20	(28.6%)		19	(24.4%)		12	(25.5%)		10	(16.4%)	
Rest of the world	5	(7.1%)		6	(7.7%)		< 3			5	(8.2%)	

For the overall study population, 31.3% were men, with no statistically significant difference across calendar periods (p-value = 0.27). The median age was 60.3 years [IQR: 45.0-69.6], with little difference across calendar periods. A stepwise decrease in the burden of chronic heart failure was identified (2001–2005: 54% and 2016–2021: 20%, p-value < 0.001), while the burden of AF seemed stable (2001–2005: 51% and 2016–2021: 46%, p-value = 0.27). Most patients had Danish origin (2001–2005: 61% and 2016–2021: 67%, p-value = 0.46). The proportion of patients with rheumatic MS was 63% in 1996–2000, 64% in 2001–2005, 55% in 2006–2010, 51% in 2011–2015, and 33% in 2016–2021 (p-value < 0.001). The incidence rate of surgical intervention decreased in patients with a different origin than Denmark, while it remained stable in patients with a Danish origin, as shown in Supplementary Table 1^24^.

### The incidence rate of surgical intervention per calendar period

**Fig. 2:**
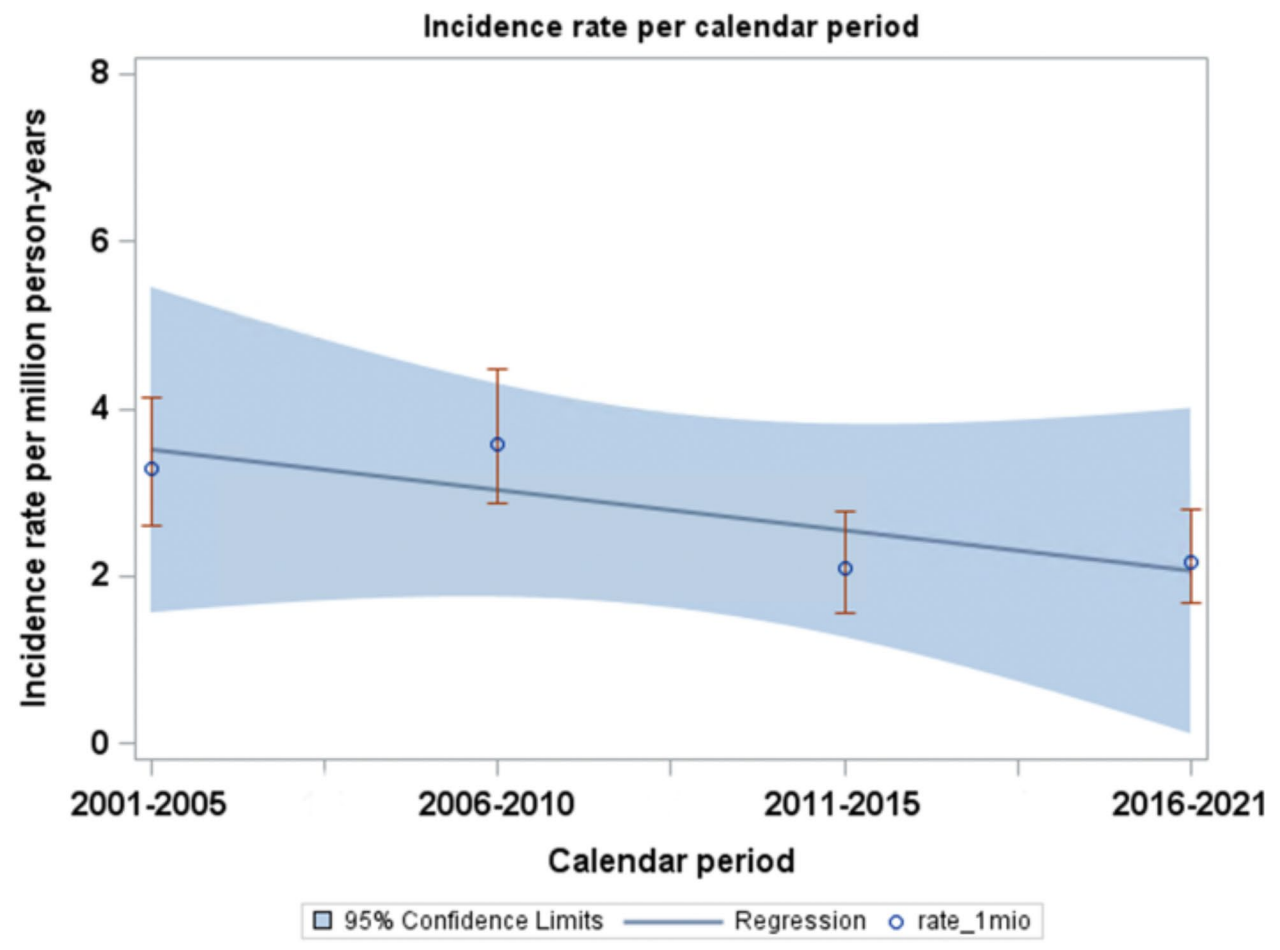
Incidence of surgical intervention for MS per calendar period. This figure shows the incidence of surgical intervention for MS per million person-years according to calendar periods.

**Fig. 3:**
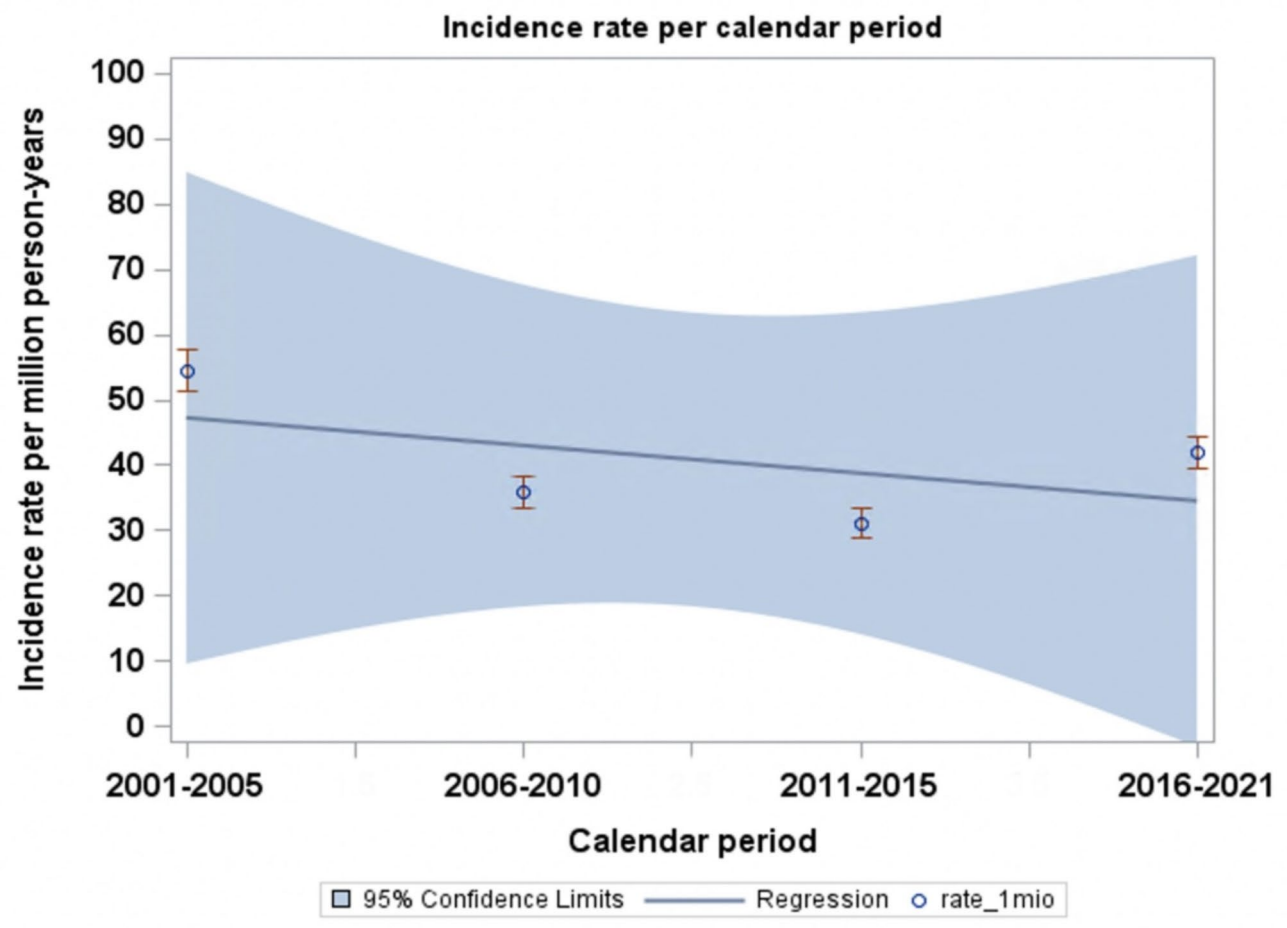
Incidence of MS per calendar period. This figure shows the incidence of MS per million person-years according to calendar periods.

The incidence rate of MS surgery steadily decreased from 3.3 incidences per million person-years in the first calendar period (2001–2005) to 2.2 incidences per million person-years in the last calendar period (2016–2021), as shown in Fig. 2. The incidence of patients diagnosed with first-time MS decreased over calendar periods (2001–2005: 54.5 incidences per million person-years, 2006–2010: 35.9, 2011–2015: 31.0, and 2016–2021: 41.9), as shown in Fig. 3. The proportion of patients undergoing surgery for MS was 2001–2005: 6.1%, 2006–2010: 10.6%, 2011–2015: 6.8%, and 2016–2021: 5.2%.

### All-cause mortality

**Fig. 4:**
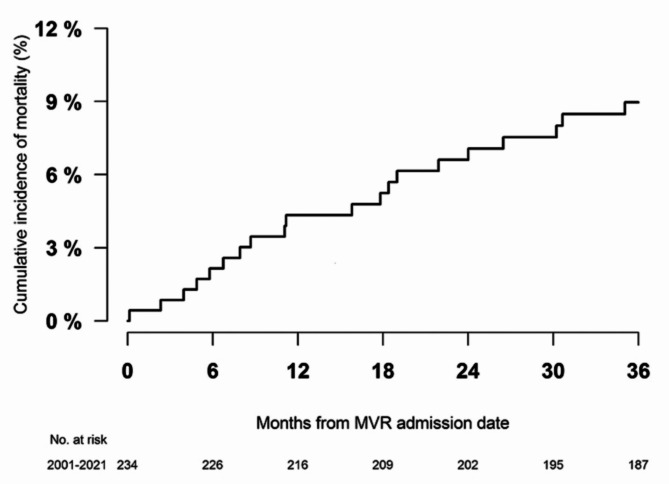
Three-year cumulative incidence of all-cause mortality. This figure shows the three-year cumulative incidence of all-cause mortality.

**Fig. 5:**
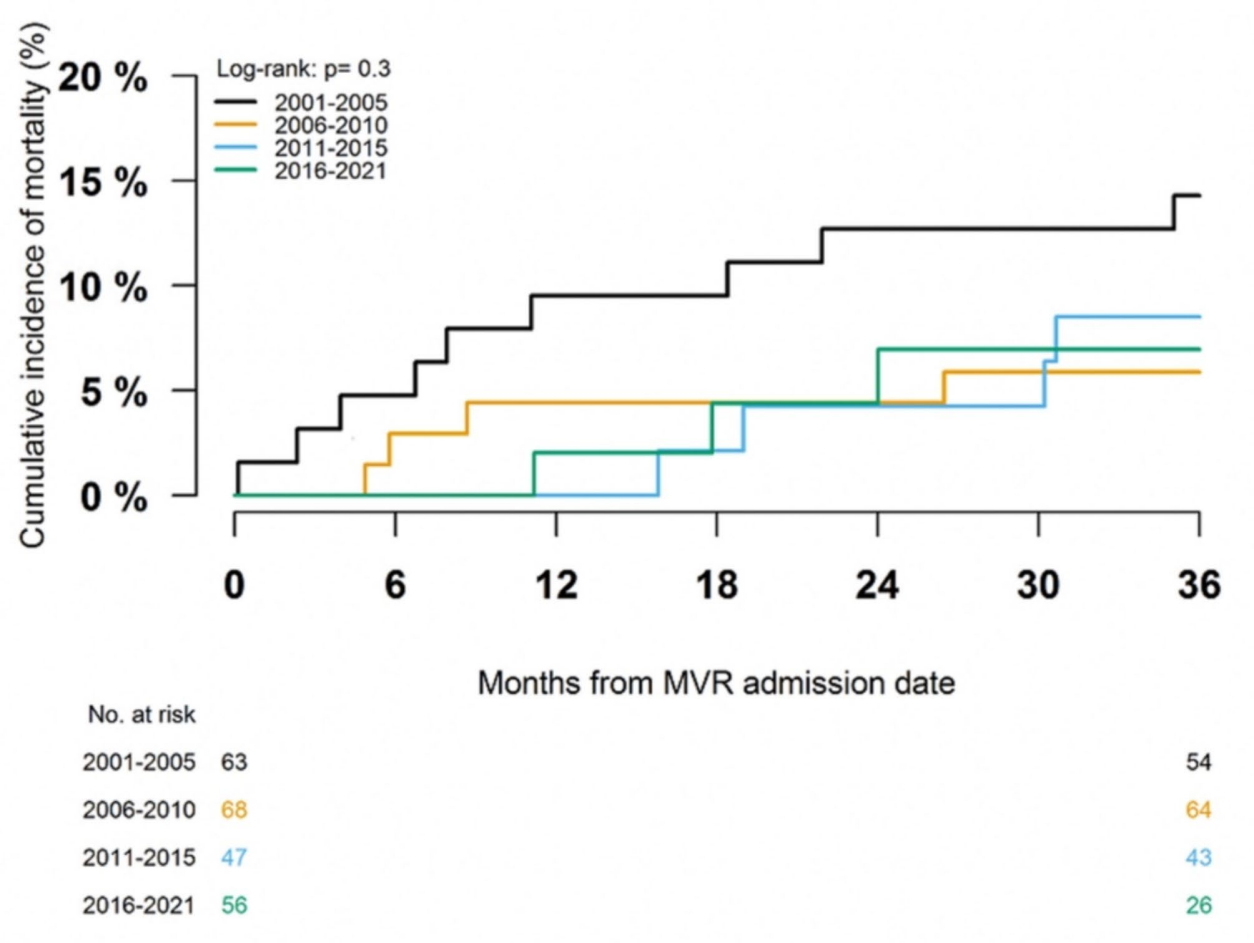
Three-year cumulative incidence of all-cause mortality according to calendar periods. This figure shows the three-year cumulative incidence of all-cause mortality according to calendar periods.

For the overall study period (2001–2021), all-cause mortality with a maximum of three years (median follow-up 2.96 years, mean follow-up of 2.43 years) after the date of surgery was 16.8%. The in-hospital mortality was 8.6%. For patients surviving in-hospital stays, the mortality was 9.0% with a maximum of three years of follow-up from surgery discharge (median follow-up 2.96 years, mean follow-up 2.67 years), as shown in Fig. 4. According to calendar periods, we identified all-cause mortality with a maximum of three years of follow-up from surgery discharge at 14.3% in 2001–2005, 5.9% in 2006–2010, 8.5% in 2011–2015, and 7.0% in 2016–2021 (*p* = 0.07 for the crude difference between groups), as shown in Fig. 5.

As compared to calendar period 2001–2005, adjusted analysis showed no statistically significant difference in associated rates of mortality across calendar periods (calendar period 2006–2010: HR 0.47 [95% CI 0.12–1.90], calendar period 2011–2015: HR 0.41 [95% CI 0.13–1.33], calendar period 2016–2021: HR 0.37 [95% CI 0.09–1.65]). Chronic obstructive pulmonary disease was associated with increased mortality (HR 3.95 [95% CI 1.47–10.58]) and chronic renal failure was associated with increased mortality (HR 3.85 [95% CI 0.96–15.45]), however statistically insignificant.

### Additional information about the surgical intervention

Data from the East Danish Database on cardiac surgery was available for 54 patients (21.1%). The left ventricular ejection fraction (LVEF) prior to surgery was recorded in 40 of these patients; 10 (25%) had a LVEF ≥ 55%, 25 (62.5%) had a LVEF of 40–54%, and 5 (12.5%) had a LVEF of 30–39%. The body mass index (BMI) prior to surgery was recorded in 52 patients; 23 (42.6%) had a BMI between 18 and 24.9, 20 (37.0%) had a BMI between 25 and 29.9, 6 (11.1%) had a BMI between 30 and 34.9, and 3 patients (5.6%) had a BMI over 35.

After surgery, 35 of the 54 patients (64.8%) experienced postoperative AF and 19 (54.3%) of the 35 patients with postoperative AF had a history of AF prior to surgical intervention. The median time of surgery in the 54 patients was 122 min [p25: 96 min and p75: 154 min].

### Sensitivity and supplementary analysis

Patients excluded due to mitral regurgitation as a primary diagnosis within a year prior to surgical intervention were compared to the study population according to patient characteristics, and we found no major differences, as shown in Supplementary Table 2. The incidence of surgical intervention in these patients was calculated per million person-years in the five different calendar periods, and it decreased from 4.9 incidences per million person-years in the first calendar period (2001–2005) to 2.3 incidences per million person-years in the last calendar period (2016–2021), as shown in Supplementary Fig. 1.

## Discussion

In this nationwide observational study with data from more than 20 years (2001–2021), we examined patient characteristics, incidence rate, and three-year mortality in patients undergoing surgery for MS. The study yielded three major findings. First, we did not find any significant changes in patient characteristics across calendar periods. Second, we found that the incidence rate of patients undergoing surgical intervention for MS decreased across calendar periods. Third, we found no statistically significant changes in mortality over calendar periods.

We found that patients undergoing surgical intervention had a median age of around 60 years and were mostly female (69%) with little differences across calendar time. This was expected, as an increased prevalence of MS in both younger and female patients is well known^13^. The burden of cardiac morbidity was high in the patients studied, as around 1 in 2 patients suffered from hypertension (51%), chronic heart failure (41%), and AF (48%). This characterization is in line with previous findings on similar cohorts, underscoring the significant burden of comorbidities in these patients^9,14–18^. Our study separates itself from the studies as it provides data from a nationwide cohort, regardless of region or center.

While we observed no major differences in patient characteristics (medical and surgical history) across calendar periods, more patients were treated with coronary artery bypass grafting and fewer patients were diagnosed with chronic heart failure in recent calendar time. On one hand, this could be caused by a decline in the burden of heart failure, but on the other hand, this could also be because of a more restrictive selection process in patients undergoing surgery.

Noteworthy was our finding that 2 in 3 patients were of Danish origin and almost 1 in 4 patients were of Asian origin. 212 patients (55.4%) had a diagnosis of rheumatic mitral valve disease prior to surgical intervention for MS, and this distribution decreased over calendar periods.

An increased associated incidence of rheumatic heart valve disease in patients of Asian and African origin has previously been shown in a Swedish nationwide study^1^. Immigration from developing countries urges clinical awareness of this valve disease, also in high-income countries.

The incidence of surgical intervention for MS decreased over calendar periods from 3.3 incidences per million person-years in the first calendar period (2001–2005) to 2.2 incidences per million person-years in the last calendar period (2016–2021). Surgical data is important in the epidemiological understanding of MS, as it sheds light on some of the most severe cases of MS. However, it must be acknowledged from the findings of our study that the data sources for this study did not provide information on patients with severe MS where surgery could not be offered.

The decrease of intervention for MS over calendar period was expected considering the incidence of rheumatic MS has generally been falling in high-income countries over several decades^9^. A resurgence in a few Western countries and areas has been observed in recent years^1–6^. Areas with a resurgence in MS incidence have also experienced a noteworthy increase in refugees. Such an increase in refugees has not happened in Denmark and could be the reason the incidence of rheumatic MS and surgical intervention for rheumatic MS have remained stable^19^. Yet this is only speculation, and an unambiguous answer remains.

We found an all-cause mortality rate of around one in six patients after three years of follow-up (including in hospital mortality). When comparing crude estimates across calendar periods, the mortality trend remained stable, which was not unexpected considering the diagnostic work-up has not changed noteworthy over this time-period. Few studies have examined death after surgical intervention for MS alone, but multiple studies investigating mortality after surgical intervention for mitral regurgitation and MS have been conducted^20–24^. These studies have reported long-term mortality rates of 13–22% for patients undergoing mitral valve replacement^20–22^ and 4–8% for patients undergoing mitral valve repair^22–24^. Our finding of all-cause mortality seems to be in between the long-term mortality for the two different types of surgical interventionbut closer to the long-term mortality after mitral valve replacement. This matches the distribution of type of surgery, as more patients in our study group underwent mitral valve replacement (75%) than mitral valve repair (25%).

Our study had some limitations. First, as this is an observational study, we cannot draw any causal conclusions but only report associations.

Second, exposure variables were defined by ICD-10 codes from nationwide registries. The PPV of the codes used for the identification of surgical interventions has previously been shown to be accurate^25^. Third, the results from our study can only be compared to populations and healthcare systems that resemble the Danish population and healthcare system, which is an industrial, high-income country. Our findings cannot be extrapolated to other geographic regions with other socioeconomic and ethnic demographics.

Fourth, we were not able to identify the etiology of MS for all patients, but it is reasonable to assume that most of the cases are caused by rheumatic heart disease^9,12^, as 64% in 2001–2005 and 33% in 2016–2021 had a diagnosis with rheumatic heart disease prior to surgery, making comparison with data on rheumatic MS eligible.

Fifth, our study is a small-scale study, and data from other countries is needed to gain the initiative to conduct larger-scale studies and decide whether our findings are solid.

Sixth, for some study groups, the numbers are small, which limits the power and increases the possibility of type II errors. Seventh, some patient characteristics were only available in a small subset of patients, which include preoperative echocardiography findings and the severity of MS as well as underlying morphology of the mitral valve, BMI, history of smoking, and postoperative AF. Further, data on valve size inserted during mitral valve replacement were not available. Applying these to the total population would have provided significant improvements to our analysis.

In this nationwide study spanning a period of over 20 years, our findings indicate that patients undergoing surgical intervention for MS were characterized by a high burden of cardiac morbidity. We observed minimal disparities across different calendar periods, except for the prevalence of chronic heart failure. The incidence rate of surgical intervention for MS decreased within the past 20 years, while all-cause mortality remained stable. This study brings contemporary and nationwide data from a high-income country on surgery for MS, however, international collaborations are needed to gain enough data to initiate large-scale studies.

## Methods

### Data sources

Every Danish citizen is provided with a unique personal identifier, enabling linkage between multiple national health registries. In this study, we linked the following registries: (1) Danish National Patient Registry (DNPR), which contains all Danish hospital admissions since 1977 and outpatient visits since 1995, with International Classification of Diseases (ICD)-8 and ICD-10 diagnosis codes and surgical procedures classified according to the Nordic Medico-Statistical Committee since 1996 (Diagnosis and procedure codes are shown in Supplementary Table 3)^26^. (2) The Danish Civil Registration System contains information on sex, vital status, migration, and birthdate^27^. (3) The Danish National Prescription Registry has records on claimed drug prescriptions since 1995 (ATC classification codes are shown in supplementary Table 4)^28^. (4) The Danish Population Registry has records on country of origin^29^. The Eastern Danish Database of Cardiac Surgery has data on cardiac invasive procedures, including pre- and postoperative information^30^. The registries are all high quality, validated, and described in detail^30–32^.

### Study population

We included patients undergoing surgical intervention for MS in Denmark from either; (1) A surgical code for mitral surgery due to MS; (2) An MS diagnosis prior to surgery. The diagnosis of MS was found from in- or outpatient visits between 2001 and 2021, including both primary and secondary diagnosis codes. Patients under 18 years of age, those diagnosed with endocarditis during admission for surgical intervention, or those diagnosed with mitral regurgitation as a primary diagnosis code within one year prior to surgery were excluded (Fig. 1). The diagnosis codes are validated with a positive predictive value (PPV) of 88–97% for mitral regurgitation and MS^31^ and 100% for mitral valve surgery^32^. The study population was grouped according to calendar periods (1: 2001–2005, 2: 2006–2010, 3: 2011–2015, and 4: 2016–2021).

In a separate analysis, we examined the incidence rate of patients diagnosed with mitral regurgitation or MS using primary and secondary diagnosis codes for MS, as shown in Supplementary Table 2.

### Covariates

Covariates for the assessment of baseline characteristics were age, sex, country of origin, and surgical and medical history prior to surgical intervention for MS. The country of origin was divided into four regions of the world; Denmark, Europe (Denmark excluded), Asia (including Middle Eastern countries but except northern Africa), and the rest of the world. Through ICD-10 codes, the proportion of patients with rheumatic MS was identified. The study population’s surgical and medical history was assessed from the DNPR as an in- or outpatient visit prior to the date of surgical intervention admission, apart from hypertension, which was defined from the use of claimed drug prescriptions that have been described and validated previously^25^. Diabetes was defined as both in- and outpatient visits or the use of claimed drug prescriptions prior to the date of surgical intervention admission. The study population’s pharmacotherapy was assessed by claimed drug prescriptions within six months prior to the date of surgical intervention admission. Patient numbers of less than three patients were not reported.

Certain information, such as body mass index at the time of surgery, ejection fraction prior to intervention, and surgical time, was only available for a fraction of the total study population using data from the East Danish database on cardiac surgery.

### Follow-up

For the assessment of mortality, patients were observed from the discharge from surgical intervention until a maximum of three years of follow-up, death, or the end of the study period (December 31, 2021), whichever came first.

### Statistics

Characteristics at baseline were compared by calendar groups. Categorical variables were shown as counts and percentages, and continuous variables as medians with interquartile ranges (IQR). The Cochran-Armitage trend test was used to examine differences in trend over calendar time for the burden of heart failure, AF, the proportion of patients with origins from Denmark, sex, and the proportion of patients with rheumatic MS. The Cochran-Armitage trend test was used to examine differences in trend over calendar time for the incidence of MS in the background population. The incidence rate of surgical intervention for MS was calculated considering the total Danish population at risk. The incidence rate was calculated with the numerator as the total cases divided by the total number of patients at risk per person per year per calendar period. Persons from the Danish population were considered at risk from the date of turning 18 years old or the date of immigration to Denmark. Patients contributed with person years up until emigration from Denmark or death. Risk time was attributed to calendar groups as previously described (1: 2001–2005, 2: 2006–2010, 3: 2011–2015, and 4: 2016–2021). The 95% confidence intervals (CI) were calculated assuming a Poisson distribution. A similar method was applied to compute the incidence rate of patients diagnosed with MS according to calendar periods.

All-cause mortality was assessed using the reverse Kaplan–Meier estimator and grouped according to calendar periods. The log-rank test was used to test differences between calendar periods.

Using multivariable-adjusted Cox proportional hazards analysis, we assessed the associated rates of outcomes. The model examining mortality included the following covariates: chronic obstructive pulmonary disease, chronic renal failure, sex, age, chronic heart failure, and malignancy. Results from the Cox regression analysis were reported with hazard ratios (HR) and 95% CI. The Cox proportional hazard’s assumption was tested using Martingale’s residuals and reported if it was violated.

All statistical analyses were made using the SAS statistical software (version 9.4, Cary, NC, USA) and R (version 3.6.1, The R Foundation, Vienna, Austria)^33^. The level of statistical significance was interpreted as a P-value of < 0.05.

### Supplementary analysis

In a supplementary analysis, we repeated analyses, including patients who underwent mitral valve replacement with a previous diagnosis code of both MS and mitral regurgitation.

### Ethics

In Denmark, register-based studies that are conducted for the sole purpose of statistics and scientific research do not require ethical approval or informed consent by law^34^. However, the study was approved by the data responsible institute (Capital Region of Denmark) with approval number P-2019-348 in accordance with the General Data Protection Regulation (GDPR)^35^. All personal identifiers were anonymized and sub classifications with three or less patients are not reported in order to assure anonymization as by rules of Statistics Denmark.

## Electronic supplementary material

Below is the link to the electronic supplementary material.

Supplementary Material 1

## Data Availability

The data underlying this article were accessed from Statistics Denmark by permission. Data will be shared on request to the corresponding author with permission of Statistics Denmark.
