# Supplementary material for: Temporal changes in characteristics, incidence, and mortality in patients undergoing surgical intervention for mitral stenosis
Source: Sci Rep. 2024 Oct 16;14:24288. doi: 10.1038/s41598-024-74807-5 (PMC11484946; doi:10.1038/s41598-024-74807-5)
Supplement: Supplementary file 1 — Supplementary Material 1 [file 41598_2024_74807_MOESM1_ESM.docx]

**Supplementary Materials**

**Supplementary table 1**

**Supplementary table 1 - This table shows the incidence rate pr million inhabitants of surgical intervention for Mitral stenosis in patients with and without Danish origin**

|  | Calendar period  2001-2005 | | Calendar period 2006-2010 | | Calendar period 2011-2015 | | Calendar period 2016-2021 | |
| --- | --- | --- | --- | --- | --- | --- | --- | --- |
|  | N | IR pr 1 million | N | IR pr 1 million | N | IR pr 1 million | N | IR pr 1 million |
| Danish origin | 43 | 8.42 | 50 | 9.81 | 31 | 6.03 | 41 | 7.90 |
| Other origin | 27 | 97.82 | 28 | 73.49 | 16 | 34.71 | 20 | 33.84 |

**Supplementary table 2**

| Supplementary Table 2. Baseline characteristics by calendar periods for patients undergoing mitral valve surgery with mitral regurgitation as a primary diagnosis within a year prior to surgery and mitral stenosis diagnosis. | | | | | | | | | | | | | | | | |
| --- | --- | --- | --- | --- | --- | --- | --- | --- | --- | --- | --- | --- | --- | --- | --- | --- |
|  | Calendar period  2001-2005  N=104 | | | Calendar period  2006-2010  N=79 | | | Calendar period  2011-2015  N=66 | | | | Calendar period  2016-2021  N=66 | | | |  |  |
| *Demographics* | | | | | | | | | | | | | | | | |
| Male sex (%) | 28 | | (26.9%) | 18 | | (22.8%) | 11 | | (16.7%) | | 19 | | (28.8%) | |  |  |
| Age median years (IQR) | 61.7 | | (49.4-68.9) | 63.0 | | (48.5-72.1) | 58.0 | | (50.4-69.0) | | 60.9 | | (53.9-71.3) | |  |  |
|  |  |  | |  |  | |  |  | |  | |  | |  | |  |
| *Duration of admission* | | | | | | | | | | | | | | | | |
| Days | 14.0 | | (10.0-25.0) | 15 | | (9.0-24.0) | 13.0 | | (9.0-29.0) | | 13.5 | | (9.0-27.0) | |  |  |
|  |  |  | |  |  | |  |  | |  | |  | |  | |  |
| *Diagnosis of rheumatic mitral valve disease prior to surgical intervention* | | | | | | | | | | | | | | | | |
|  | 70 | | (67.3%) | 43 | | (54.4%) | 35 | | (53.0%) | | 30 | | (45.5%) | |  |  |
|  |  |  | |  |  | |  |  | |  | |  | |  | |  |
| *Type of mitral valve surgery* | | | | | | | | | | | | | | | | |
| Repair | 0 | | (0%) | 0 | | (0%) | 0 | | (0%) | | 0 | | (0%) | |  |  |
| Replacement | 104 | | (100%) | 79 | | (100%) | 66 | | (100%) | | 66 | | (100%) | |  |  |
|  |  |  | |  |  | |  |  | |  | |  | |  | |  |
| *Balloon mitral valvotomy* | | | | | | | | | | | | | | | | |
|  | 0 | | (0%) | 0 | | (0%) | 0 | | (0%) | | 0 | | (0%) | |  |  |
|  | | | | | | | | | | | | | | | | |
| *Other heart surgery performed on the day of MS surgery* | | | | | | | | | | | | | | | | |
| CABG | 15 | | (14.4%) | 9 | | (11.4%) | 5 | | (7.6%) | | 4 | | (6.1%) | |  |  |
| Aortic valve surgery | 21 | | (20.2%) | 16 | | (20.3%) | 14 | | (21.2) | | 11 | | (16.7%) | |  |  |
| Tricuspid valve surgery | 4 | | (3.9%) | 9 | | (11.4%) | 8 | | (12.1%) | | 12 | | (18.2%) | |  |  |
|  | | | | | | | | | | | | | | | | |
| *Prior cardiac surgery* | | | | | | | | | | | | | | | | |
|  | <3 | |  | 3 | | (3.8%) | <3 | |  | | 6 | | (9.1%) | |  |  |
|  | | | | | | | | | | | | | | | | |
| *Other heart valve diseases* | | | | | | | | | | | | | | | | |
| Aortic regurgitation | 25 | | (24.0%) | 18 | | (22.8%) | 14 | | (21.2%) | | 21 | | (31.8%) | |  |  |
| Aortic stenosis | 20 | | (19.2%) | 13 | | (16.5%) | 10 | | (15.2%) | | 10 | | (15.2%) | |  |  |
|  | | | | | | | | | | | | | | | | |
| *Comorbidities* | | | | | | | | | | | | | | | | |
| Stroke | 12 | | (11.5%) | 7 | | (8.9%) | 7 | | (10.6%) | | 8 | | (12.1%) | |  |  |
| Bleeding | 16 | | (15.4%) | 9 | | (11.4%) | 16 | | (24.2%) | | 10 | | (15.2%) | |  |  |
| Heart failure | 50 | | (48.1%) | 30 | | (38.0%) | 27 | | (40.9%) | | 20 | | (30.3%) | |  |  |
| Hypertension | 60 | | (57.7%) | 43 | | (54.4%) | 31 | | (47.0%) | | 41 | | (62.1%) | |  |  |
| Ischaemic heart disease | 23 | | (32.9%) | 28 | | (35.9%) | 18 | | (38.3%) | | 17 | | (27.9%) | |  |  |
| Atrial fibrillation | 62 | | (59.6%) | 44 | | (55.7%) | 37 | | (56.1%) | | 38 | | (57.6%) | |  |  |
| COPD | 13 | | (12.5%) | 13 | | (16.5%) | 16 | | (24.2%) | | 13 | | (19.7%) | |  |  |
| Liver disease | <3 | |  | <3 | |  | 6 | | (9.1%) | | 4 | | (6.1%) | |  |  |
| Chronic renal failure | 5 | | (4.8%) | 3 | | (3.8%) | 10 | | (15.2%) | | 9 | | (13.6%) | |  |  |
| Diabetes | 7 | | (6.7%) | 16 | | (20.3%) | 9 | | (13.6%) | | 15 | | (22.7%) | |  |  |
| Malignancy | 6 | | (5.8%) | 11 | | (13.9%) | 6 | | (9.1%) | | 12 | | (18.2%) | |  |  |
|  | | | | | | | | | | | | | | | | |
| *Medications* | | | | | | | | | | | | | | | | |
| Calcium channel blockers | 11 | | (10.6%) | 6 | | (7.6%) | 11 | | (16.7%) | | 16 | | (24.2%) | |  |  |
| RAS inhibitors | 27 | | (26.0%) | 26 | | (32.9%) | 14 | | (21.2%) | | 24 | | (36.4%) | |  |  |
| Loop diuretics | 60 | | (57.7%) | 44 | | (55.7%) | 38 | | (57.6%) | | 42 | | (63.6%) | |  |  |
| Aspirin | 22 | | (21.2%) | 23 | | (29.1%) | 21 | | (31.8%) | | 10 | | (15.2%) | |  |  |
| OAC | 67 | | (64.4%) | 51 | | (64.6%) | 36 | | (54.5%) | | 42 | | (63.6%) | |  |  |
| Statin | 14 | | (13.5%) | 28 | | (35.4%) | 27 | | (40.9%) | | 31 | | (37.0%) | |  |  |
| Beta blockers | 41 | | (39.4%) | 34 | | (43.0%) | 28 | | (42.4%) | | 30 | | (45.5%) | |  |  |
| Digoxin | 46 | | (44.2%) | 28 | | (35.4%) | 17 | | (25.8%) | | 13 | | (19.7%) | |  |  |
|  | | | | | | | | | | | | | | | | |
| *Geographic region* | | | | | | | | | | | | | | | | |
| Denmark | 84 | | (80.8%) | 49 | | (62.0%) | 45 | | (68.2%) | | 45 | | (68.2%) | |  |  |
| Europe (DK excluded) | 4 | | (3.9%) | 9 | | (11.4%) | 3 | | (4.6%) | | 4 | | (6.1%) | |  |  |
| Asia | 14 | | (13.5%) | 15 | | (19.0%) | 16 | | (24.2%) | | 15 | | (22.7%) | |  |  |
| Rest of the world | <3 | |  | 6 | | (7.6%) | <3 | |  | | <3 | |  | |  |  |

**Supplementary Table 3: Diagnosis and procedure codes**

|  | **ICD-8 code(s)** | **ICD-10 code(s)** | **Procedure codes*** |
| --- | --- | --- | --- |
| Acute myocardial infarction | 410 | I21, I22 | N/A |
| Ischemic heart disease | 410-414 | I20-I25 | N/A |
| Heart failure | 425, 428, 4270, 4271 | I42, I50, I110, I130, I132, J819 | N/A |
| Ventricular arrythmia |  | DI47, DI49 | N/A |
| Ischemic stroke | 430-433, 434, 436 | I60-I64 | N/A |
| Peripheral vascular disease | 440, 444 | I70, I74 | N/A |
| Chronic obstructive pulmonary disease | 490-492 | J42-J44 | N/A |
| Liver disease | 070, 155, 571-573 | B15-B19, C22, D684C, K70-K77, I982, Q618A, Z944 | N/A |
| Malignancy | 140-209 | C00-C97 | N/A |
| Diabetes** | 250 | E10-E14 | N/A |
| Bleeding |  | I60-I62, N02, R31,R04, D62, H052A, G951A, S368D, K298A, K228F, I864A, K638B, K638C, K638F,K868G, I312, H313, H356, H431, H450, S064-S066, J942, D500, K250, K252, K254, K256, K260, K262, K264, 266, K270, K272, K274, K276, K280, K282,K284, K286, K290, K625, K661, K920, K921, K922, I850 | N/A |
| Alcohol | 291, 303, 57109, 57110, 57710 | F10, K70, E52,T51, K860, E244, G312, I426, O354, Z714, G621, G721, K292, L278A | N/A |
| Mitral Stenosis | 39400, 39490 | I050, I342 | N/A |
| Mitral regurgitation | 39491 | I340, I34I, I052 | N/A |
| Aortic Regurgitation | 39501, 39592 | I351 | N/A |
| Aortic Stenosis | I060, I350, 39500 | I350 | N/A |
| Balloon mitral valvotomy |  |  | KFKA32 |
| Mitral valve repair | N/A | N/A | KFKC00, KFKC10, KFKC30, KFKC40, KFKC60, KFKC96, KFKH10, KFKB10, KFKB96, KFKB00 |
| Mitral valve replacement | N/A | N/A | KFKD00, KFKC20, KFKC50, KFKD51, KFKD96, KFKD1, KFKD20, KFKD54 |
| Aortic valve surgery | N/A | N/A | KFM |
| Tricuspid valve surgery | N/A | N/A | KFG |
| Coronary artery bypass graft surgery | N/A | N/A | KFNA,KFNB, KFNC,KFND, KFNE |

*Abbreviations: ICD-8, International Classification of Diseases, 8^th^ revision; ICD-10, International Classification of Diseases, 10^th^ revision; N/A, not applicable.*

** According to the Nordic Medico-Statistical Committee Classification of Surgical Procedures.*

*** or patients with a filled-in prescription of antidiabetics within 6 months of MR diagnosis (ATC-code: A10)*

**Supplementary Table 4: ATC classification codes**

| **Pharmacotherapy** | **ATC code(s)** |
| --- | --- |
| Beta-blockers* | C07, C09BX |
| Calcium channel blockers* | C07F, C08, C09BB, C09DB |
| Renin-angiotensin system inhibitors* | C09 |
| Vasodilator drugs* | C02DB, C02DD, C02DG |
| Antiadrenergic drugs* | C02A, C02B, C02C |
| Thiazides* | C03A, C07B, C07D, C09XA52, C03EA01 |
| Loop diuretics* | C03C, C03EB01, C03EB02 |
| Mineralocorticoid receptor antagonists* | C03DA01-C03DA04 |
| Anti-diabetics | A10 |
| Aspirin | B01AC06 |
| ADP receptor inhibitors | B01AC04, B01AC06, B01AC22, B01AC24 |
| Statins | C10AA |
| Oral anticoagulants | B01AA, B01AF, B01AE |
| Digoxin | C01AA05 |
| Amiodaron | C01BD01 |

*Abbreviations: ATC, Anatomical Therapeutic Chemical; ADP, adenosine diphosphate.*

**Hypertension was defined by claimed prescriptions of two or more of the marked antihypertensive medications within six months prior to index.*

**Supplementary figure 1**


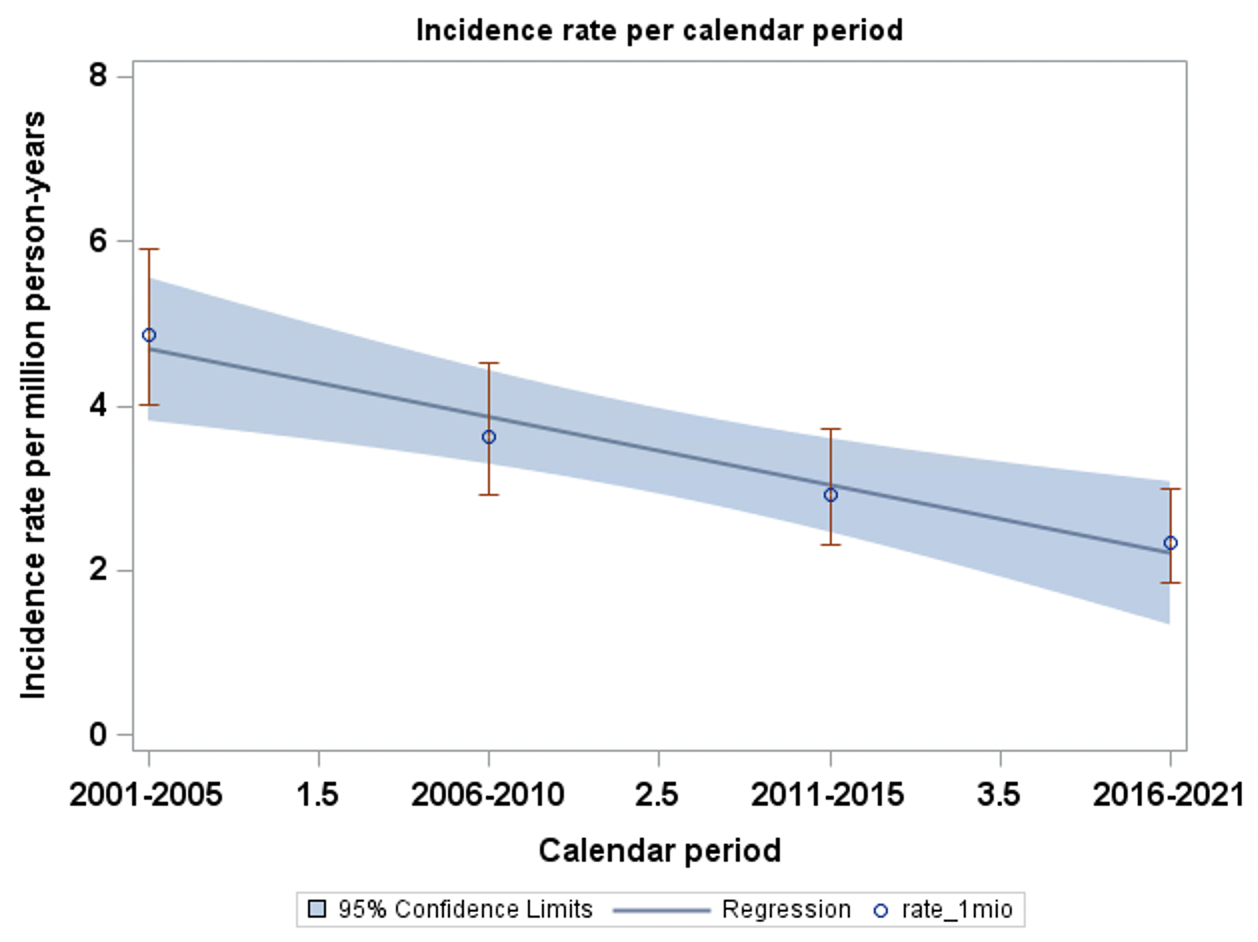

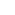

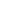

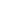

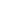

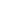

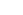

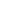

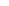

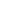

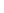

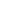

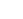

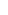

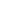

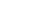

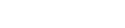


This figure shows the incidence of surgical intervention per million person-years according to calendar periods in patients with mitral stenosis and with mitral regurgitation as a primary diagnosis within a year prior to surgery.
